# *Mycoplasma Pneumonia*: An Unrecognized Cause of Fever of Unknown Origin in an Adult

**DOI:** 10.1155/2017/6854913

**Published:** 2017-10-17

**Authors:** Fatima Ali-Ahmed, Alexandra Halalau

**Affiliations:** ^1^Internal Medicine/Pediatrics Department, Beaumont Health, Royal Oak, MI, USA; ^2^Internal Medicine Department, General Internal Medicine Division, Beaumont Health, Royal Oak, MI, USA; ^3^Oakland University William Beaumont School of Medicine, Rochester, MI, USA

## Abstract

A 26-year-old female was admitted for fever of unknown origin (FUO), headaches, left ankle edema, and a lower extremity rash consistent with erythema nodosum. She had no respiratory symptoms or family history of autoimmune diseases. A chest X-ray was negative for pneumonia or hilar adenopathy. Extensive autoimmune workup was negative. A chest, abdomen, and pelvis computed tomography scan was unremarkable and laboratory studies revealed no source of infection. On hospital day 5, the patient developed a mild productive cough. Her* Mycoplasma pneumonia* (MP) IgM was high, confirming the diagnosis of MP induced FUO. She was started on azithromycin 500 mg daily and within 24 hours her fevers and headaches resolved. Her left ankle edema and EN gradually improved over a course of a few weeks. This case report highlights the need for MP testing in the evaluation of fever of unknown origin, even in the absence of pulmonary manifestations.

## 1. Background

*Mycoplasma pneumonia* (MP) is a small pathogenic bacteria that generally infects pulmonary tissue. MP is well known to cause a wide spectrum of extrapulmonary manifestations including neurological, cardiac, and dermatological (among several others) disorders [1, 2]. The disease is transmissible via aerosols. Particularly vulnerable populations are children and immunocompromised persons [1, 2].

MP is an obligate parasite. Due to its lack of a rigid cell wall, it can fuse with or invade mucosal membranes of epithelial cells and impair the efficacy of beta-lactam antibiotics [2]; therefore, a diagnosis is crucial because empiric beta-lactam antibiotics used to treat community acquired pneumonia are ineffective against* MP* [2]. Currently, enzyme immunoassays (EIAs) IgM and IgG titers are the most common and reliable* Mycoplasma* serology tests used [2]. MP is difficult to diagnose because of weak IgM antibodies responses in a reinfected adult [3]. Thus, an increase in IgM and IgG titers in the convalescent phase indicates a current or recent infection [2, 3]. However, in 25% of infections, single acute-phase serology, IgM base-EIA, can be diagnostic for MP infection [4].

Another serious manifestation of MP is erythema nodosum (EN) [5, 6]. EN is a painful dermal inflammatory condition, typically confined to the patient's shins. While 35–55% of EN cases are idiopathic, EN is also associated with autoimmune diseases, neoplasms, and some infection types [6].

The differential diagnosis of FUO is vast and challenging, with an etiology list of over 200 diseases [7]. In 10–50% of the cases, the diagnosis remains unknown [8]. A generally accepted definition of FUO is a fever of ≥101°F (38.3°C) or higher, lasting for three or more weeks, with no definitive underlying pathology after three outpatient visits or three inpatient days [7]. The diagnostic workup is directed by a thorough history and physical examination, in order to identify subtle “potential diagnostic clues,” rather than the more time-consuming “running through the list” approach [8].

To our knowledge, we present the first adult case of MP presenting as FUO with erythema nodosum without respiratory symptoms or lung involvement.

## 2. Case Presentation

A 26-year-old African American female, previously healthy, presented with a 2.5-week history of high-grade fevers and headaches. Two weeks prior to the onset of her fevers, she complained of upper respiratory symptoms, which completely resolved within a few days. The patient reported fevers ranging from 38.4°C to 39.3°C (101.1°–102.7°F) with the highest fevers occurring at night. Her fever would respond to ibuprofen and acetaminophen, but would not completely resolve. She also complained of anorexia, chills, sweats, fatigue, and a constant headache associated with photophobia and nausea, but no neck tenderness. Three days prior to admission, she developed a tender skin rash over her shins and left ankle swelling. She denied recent travel, chest pain, shortness of breath, other joint pain, oral ulcers, abdominal pain, or a family history of autoimmune disease.

**Figure 1:**
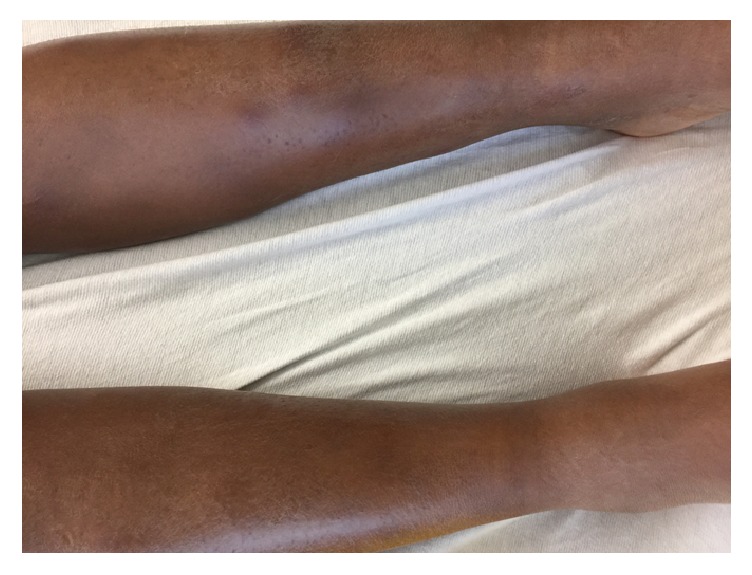
Erythema nodosum on pretibial surface of lower extremity.

On presentation, her vital signs revealed a temperature of 38.4°C (101.1°F), heart rate of 113 bpm, blood pressure of 104/53 mmHg, and a respiratory rate of 18 bpm. The patient appeared acutely ill, but she was alert and oriented ×3. She had no meningeal signs; pupils were equal, round, and reactive to light; however she did have photophobia. Breath sounds were clear to auscultation, with no wheezing or crackles. She had left ankle tenderness to palpation associated with edema, but no joint rigidity. Skin examination revealed multiple bilateral tender subcutaneous nodules on her anterior shins, consistent with erythema nodosum (EN) (Figure 1). The remainder of her exam was unremarkable, including no murmurs, lymphadenopathy, or organomegaly. Her symptoms of fever and headache raised a suspicion for meningitis. She underwent a head CT, which was unremarkable, followed by a lumbar puncture. The patient was empirically started on vancomycin and piperacillin-tazobactam.

Laboratory findings (Table 1) showed a normal total leukocyte count of 6.3 bil/L, mild normocytic anemia, elevated ESR 27, CRP 7.7, and negative CSF studies. A chest X-ray was negative for pneumonia and hilar adenopathy. After 24 hours of vancomycin and piperacillin-tazobactam, the patient continued to spike fevers of 38.9–39.4°C (102.1°–102.9°F) with no source of bacterial infection identified. Her EN and joint pain were concerning for an autoimmune process, particularly acute sarcoidosis (Lofgren syndrome); therefore antibiotics were discontinued and she was started on ibuprofen 800 mg every 8 hours.

## 3. Investigations

A respiratory viral panel was positive for Human Rhinovirus but did not explain the patient's prolonged high-grade fevers and likely represents her previous upper respiratory symptoms 2 weeks prior to the onset of her fevers. In addition, an EBV panel was positive for EBV IgG, EBV early Ag, and EBV Nuclear Ag, but IgM was negative, representing a previous infection. Also, the patient denied any symptoms of infectious mononucleosis (IM), such as pharyngitis and myalgia, and had no physical exam findings consistent with IM, such as lymphadenopathy or splenomegaly.

A chest, abdomen, and pelvis computed tomography (CT) scan revealed no hilar adenopathy, no source of an infection, and no malignancy process. An EKG revealed normal sinus rhythm with nonspecific T wave abnormalities and a 2D echo revealed an estimated ejection fraction of 40–45%. However, a cardiac magnetic resonance imaging (MRI) showed no evidence of depressed LV function with an estimated ejection fraction of 57.59%. Interestingly, the cardiac MRI reported an infiltrative disease of the left ventricular septum concerning for possible acute sarcoidosis. There was no evidence of delayed enhancement to suggest cardiac fibrosis or chronic granulomatous disease. The patient underwent a positron emission tomography (PET) cardiac sarcoidosis protocol for further evaluation, which revealed no concern for sarcoid myocardial invasion. At this point, acute sarcoidosis (Lofgren syndrome) was ruled out.

On day five of admission, the patient continued to have high-grade fevers. An extensive autoimmune workup was negative and the infectious workup (Table 1) revealed no source. She complained of worsening EN extending to her thighs, a mild productive cough, and a persistent headache. Breath sounds were still clear to auscultation with no crackles. She underwent brain MRI, which was normal. A repeat CRP increased to 12.3 from 7.7 since admission. The combination of cough and EN, and the fact that* Mycoplasma pneumonia* is a known entity of FUO in the pediatric population prompted the investigation of MP. Surprisingly, on day 7 of her admission, her MP EIA IgM and IgG titers returned positive, 1.96 and 2.10 (>1.09 positive), confirming our diagnosis of MP as the cause of her FUO with dermatological involvement prior to pulmonary manifestations.

The patient was started on azithromycin 500 mg daily and within 24 hours (hospital day 8) her fever and headache resolved. She was treated with a ten-day course of azithromycin 500 mg. Her left ankle edema and EN gradually improved over a few weeks. Months later, the patient remains afebrile with no further episodes of joint pain or EN.

## 4. Discussion

MP is an intracellular pathogen, commonly responsible for respiratory tract diseases. 25% of infected individuals with MP experience extrapulmonary manifestations before, after, during, or even in the absence of respiratory symptoms [1, 2, 5]. MP pneumonia and extrapulmonary manifestations are independent matters in which independent pathomechanisms are involved [9]; thus extrapulmonary manifestations do frequently occur in the absence of pneumonia. Although the pathomechanisms of extrapulmonary diseases remain largely unknown, the proposed theories of skin and mucosal involvement include immune complex-mediated vascular injury, cell-mediated immune response and cytotoxic injury to epithelial cells, and autoimmune mechanisms [1, 2]. However, MP has been identified by PCR and by culture in extrapulmonary sites such as skin lesions; therefore, a direct invasion must be considered with or without pulmonary involvement [1, 2, 5].

MP pneumonia develops via immune pathogenesis following the initial droplet infection to the lower respiratory tract [10]. MP begins to propagate on the respiratory surface of the ciliated epithelium, thereby inducing various cytokines [10]. This local host immune response occurring on the respiratory surface serves as a kind of firewall to prevent dissemination of the organism beyond the respiratory tract [10]. Therefore, extrapulmonary manifestations due to MP infection often occur in the absence of pneumonia or respiratory symptoms [10], which may be the reason for underrecognizing MP extrapulmonary manifestations. For this reason, upper respiratory tract (i.e., pharyngeal swabs) molecular detection or culture methods maybe inadequate for diagnosing extrapulmonary manifestations [9, 10]. Therefore, the diagnosis should be done by serological methods [9, 10].

Our patient initially manifested fever of unknown origin, headaches, and erythema nodosum caused by* Mycoplasma pneumonia,* without any pulmonary symptoms. To our knowledge this is the first case in the adult population reporting FUO secondary to MP. In the pediatric populations, MP is a known cause of FUO [11]. In one pediatric case series investigating FUO, 25% of juveniles without pulmonary manifestations were positive for MP [11]. This may stem from MP being an unrecognized cause of FUO, steering physicians away from potential diagnoses. Interestingly, in juveniles, the most common presenting symptom was headache [11], similar to our patient. Due to the complexity of FUO diagnosis, an elaborate diagnostic workup is recommended, depending on the presence or absence of potential diagnostic clues, including testing for specific infections [7, 8]. To date, MP is not specifically mentioned under the list of infectious causes for FUO [7, 12]. Therefore, we contend that MP should be included in the evaluation of FUO in adults, even in the absence of pulmonary manifestations.

## Figures and Tables

**Table 1:** Laboratory findings during admission. ANA: antinuclear antibody, Anti-dsDNA: Anti-Double Stranded DNA, ANCA: antineutrophil cytoplasmic antibody, Ab: antibody, C: complement, RNP: ribonucleoprotein, CCP: anticyclic citrullinated peptide, ENP: extractable nuclear antibodies, RF: rheumatoid Factor, CG: *Chlamydia trachomatis*, *Neisseria gonorrhoeae*, CMV: cytomegalovirus, HBsAg: Hepatitis B surface antigen, HCV: Hepatitis C virus. ^*∗*^Labs on admission.

*Chemistry* ^*∗*^	
Sodium	134 mmol/L
Potassium	4.5 mmol/L
Chloride	99 mmol/L
CO_2_	21 mmol/L
BUN	8 mg/dL
Creatinine	0.94 mg/dL
Albumin	3.7 g/dL
ALP	72 U/L
AST	20 U/L
ALT	11 U/L
Bilirubin total	0.7 mg/dL
*Peripheral blood* ^**∗**^	
WBC	6.3 bil/L
RBC	3.38 bil/L
Hb	10.2 bil/L
Hematocrit	30.6%
MCV	90 fL
MCHC	33 g/dL
RDW SD	41 fL
Platelet	272 bil/L
Neutrophils	4.4 bil/L
*CSF studies*	
Glucose, CSF	67 mg/dL
Protein, CSF	25 mg/dL
Color	Colorless
Clarity	Clear
WBC	1 mcL
RBC	2 mcL
CSF differential	100
RBC	94
PMN	0
Mononuclear cells	6
*Immunology*	
ANA screen	(−)
Anti-dsDNA	(−)
ANCA	(−)
Smith Ab	(−)
RNP Ab	(−)
CCP	(−)
C3	40 mg/dL
	(80–200)
C4	33 mg/dL
	(12–43)
ENP	(−)
HIV-1 RNA quantitation	<20
RF	10 IU/mL
	(0–14)
*Microbiology/virology/serology*	
GC	(−)
CMV DNA quantitation, PCR	(−)
HIV 1/2 Testing algorithm	Nonreactive
HIV-1 RNA log⁡10	<1.30
HIV-1 RNA quantitation	<20
HBsAG	(−)
HCV	(−)
QuantiFERON TB	(−)
VDRL	Nonreactive
*Cultures*	
Blood	(−)
CSF	(−)
Urine	(−)

## References

[1] Waites K. B., Talkington D. F. (2004). Mycoplasma pneumoniae and its role as a human pathogen. *Clinical Microbiology Reviews*.

[2] Kashyap S., Sarkar M. (2010). Mycoplasma pneumonia: clinical features and management. *Lung India*.

[3] Beersma M. F. C., Dirven K., Van Dam A. P., Templeton K. E., Claas E. C. J., Goossens H. (2005). Evaluation of 12 commercial tests and the complement fixation test for Mycoplasma pneumoniae-specific immunoglobulin G (IgG) and IgM antibodies, with PCR used as the "gold standard". *Journal of Clinical Microbiology*.

[4] Talkington D. F., Shott S., Fallon M. T., Schwartz S. B., Thacker W. L. (2004). Analysis of eight commercial enzyme immunoassay tests for detection of antibodies to *Mycoplasma pneumoniae* in human serum. *Clinical and Diagnostic Laboratory Immunology*.

[5] Sánchez-Vargas F. M., Gómez-Duarte O. G. (2008). Mycoplasma pneumoniae—an emerging extra-pulmonary pathogen. *Clinical Microbiology and Infection*.

[6] Schwartz R. A., Nervi S. J. (2007). Erythema nodosum: a sign of systemic disease. *American Family Physician*.

[7] Mulders-Manders C., Simon A., Bleeker-Rovers C. (2015). Fever of unknown origin. *Clinical Medicine*.

[8] Unger M., Karanikas G., Kerschbaumer A., Winkler S., Aletaha D. (2016). Fever of unknown origin (FUO) revised. *Wiener Klinische Wochenschrift*.

[9] Narita M. (2010). Pathogenesis of extrapulmonary manifestations of Mycoplasma pneumoniae infection with special reference to pneumonia. *Journal of Infection and Chemotherapy*.

[10] Narita M. (2011). Mycoplasma pneumoniae as an under-recognized agent of vasculitic disorders. *Advances in the Etiology, Pathogenesis and Pathology of Vasculitis*.

[11] Jayantha U. K. (2006). Mycoplasma pneumoniae: an unrecognized cause of pyrexia of unknown origin. *Sri Lanka Journal of Child Health*.

[12] Hersch E. C., Oh R. C. (2014). Prolonged febrile illness and fever of unknown origin in adults. *American Family Physician*.

